# Atraumatic splenic rupture, an underrated cause of acute abdomen

**DOI:** 10.1007/s13244-016-0500-y

**Published:** 2016-05-18

**Authors:** Massimo Tonolini, Anna Maria Ierardi, Gianpaolo Carrafiello

**Affiliations:** Department of Radiology, “Luigi Sacco” University Hospital, Via G.B. Grassi 74, 20157 Milan, Italy; Interventional Radiology - Department of Radiology, University of Insubria, Viale Borri 57, 21100 Varese, Italy

Dear Sir,

We recently read with interest the pictorial review of splenic emergencies by Unal et al., just published in Insights into Imaging. After describing the common injuries from blunt abdominal trauma, this paper comprehensively describes several more or less unusual non-traumatic acute conditions including splenic infarction, aneurysms and pseudoaneurysms, arterial and venous thrombosis, splenic torsion, sequestration in sickle-cell anaemia, infections, and abscesses, with an appropriate emphasis on the mainstay role of multidetector computed tomography (CT) [1].

On the basis of our personal experience, we strongly agree with the Authors when they state that the spleen is an underrated cause of acute abdomen, and that severe morbidity and mortality result from delayed or missed diagnosis of splenic lesions. However, upon finishing reading the article we thought that general radiologists should be well aware of the condition known as life-threatening atraumatic splenic rupture (ASR), which has not yet been presented [1–3].

ASR is uncommon but not exceptional: in fact, searching the English-language literature using PubMed yields more than a thousand publications, over 300 in the last ten years, the vast majority being case reports. The incidence, mechanisms, treatment guidelines, and prognosis are poorly defined due to heterogeneity and limited availability of comprehensive reviews. ASR may occur in a wide age range, from teenagers and young people (particularly from infectious causes) to the elderly. The predominant manifestations include variable degrees of upper or left-sided abdominal pain, tachycardia, and hypotension, followed at a later stage by malaise, vomiting, generalised abdominal tenderness and peritonism, and progressive haemodynamic shock [4–8].

The vast majority (over 90 %) of cases are “pathologic” ASRs, which develop in a diseased spleen from the ample but specific range of disorders listed in Table 1. Infections, coagulopathy, and neoplasms represent the three major aetiologic groups. Interestingly, a recent review of 613 cases disclosed that ASR represents the initial manifestation of the previously unknown underlying disease in over 50 % of patients [7, 8]. Alternatively, “idiopathic” ASR occasionally occurs in a normal-appearing spleen without predisposing factors [4–8].

**Table 1:** Causes of pathologic atraumatic splenic rupture

Abnormal coagulation	Therapeutic anticoagulation
	- Heparin
	- Oral warfarin, rivaroxaban
	- Systemic tissue plasminogen activator (tPA) thrombolysis
	Idiopathic thrombocytopenic purpura
	Platelet deficiencies
	Uremia - haemodialysis
Infections	Malaria
	Mononucleosis from Epstein–Barr virus infection
	Endocarditis
	Human immunodeficiency virus (HIV) infection
	Cytomegalovirus (CMV) infection
	Typhoid fever
	Babesiosis
	Dengue fever
Non-infectious inflammatory disorders	Systemic lupus erythematosus
	Polyarteritis nodosa
Haematologic malignancies	Acute and chronic myelogenous leukaemia
	Acute lymphoblastic leukaemia
	Waldenstrom’s disease
Solid malignancies	Non-Hodgkin lymphoma
	Hodgkin’s disease
	Splenic metastases
	Splenic angiosarcoma
Miscellaneous	Pregnancy
	Acute pancreatitis
	Vascular Ehlers-Danlos Syndrome
	Amyloidosis
	Ruptured benign splenic lesions (cyst, infarction, hamartoma, hemangioma, peliosis)

Due to its prevalence, malaria (particularly from *Plasmodium vivax* infection) represents the single major cause of ASR worldwide. Due to tourism, migrations, and drug resistance, malaria is increasingly encountered even outside tropical-subtropical Asia and Africa and the endemic American regions. In Western countries, malarial ASR should be suspected in non-immune returning travelers, expatriates, or recent immigrants from endemic places—even despite appropriate prophylaxis or during antimalarial therapy—and is associated with a non-negligible mortality (22 %) [9, 10]. In the setting of suspected or proven malaria, cross-sectional imaging with CT allows differentiation of the common splenic infarction from rupture, since the latter may require immediate or delayed splenectomy [11].

**Fig. 1:**
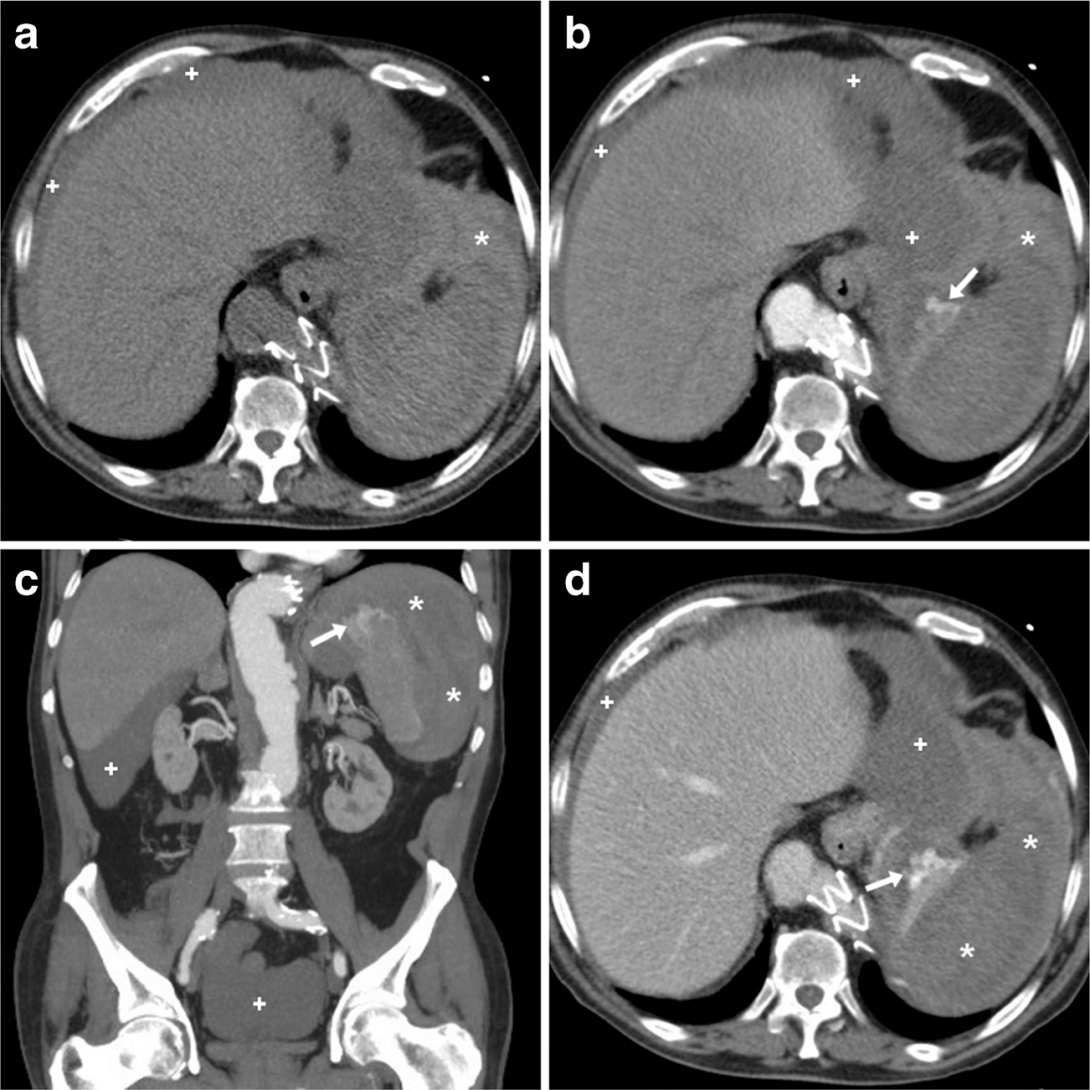
A 75-year-old male presented to emergency department with generalised acute abdominal pain and physical signs of haemodynamic impairment. His past medical history included coronary stenting and endovascular treatment of aneurysmal dilatation of the thoracic aorta, respectively, 13 and 5 years earlier. He denied trauma and unusual efforts, and was on regular warfarin anticoagulation. Bedside ultrasound (not shown) revealed echogenic peritoneal effusion. Emergency multidetector CT including unenhanced (**a**), arterial- (**b**, **c**), and portal venous (D) post-contrast acquisitions showed a normal-sized spleen, compressed by the extensive perisplenic haemorrhage (*). Contrast extravasation isoattenuating with enhanced blood vessels (arrows in B…D) and increasing from the arterial to the venous acquisition was noted at the upper splenic pole, indicating non-contained active bleeding. Multi-compartmental haemoperitoneum (+) was present. Note the metallic endoprosthesis of the distal thoracic descending aorta. Immediate splenectomy confirmed bleeding from a polar laceration. Gross and microscopic pathology did not disclose underlying abnormalities

During the last eight years at our two hospitals we encountered at least 12 cases of ASR, half of them secondary to anticoagulation (Fig. 1). In the literature, drug-related cases account for up to one-third (9-33 %) of ASRs. In the anticoagulated population, splenic bleeding is a rare complication compared to the common haemorrhages involving the anterior abdominal wall and iliopsoas muscles [12–14].

Unlike hepatic and renal hemorrhages, splenic bleeding is generally associated with diffusely infiltrated parenchyma, rather than with the presence of a solitary mass. Splenic lesions have unspecific, overlapping imaging features. However, “pathologic” ASR from neoplastic causes should be suggested in the presence of diffuse splenic infiltration or multifocal hypoattenuating lesions with mild or heterogeneous contrast enhancement, particularly in patients with known lymphomatous disease or solid tumours [8, 15].

**Fig. 2:**
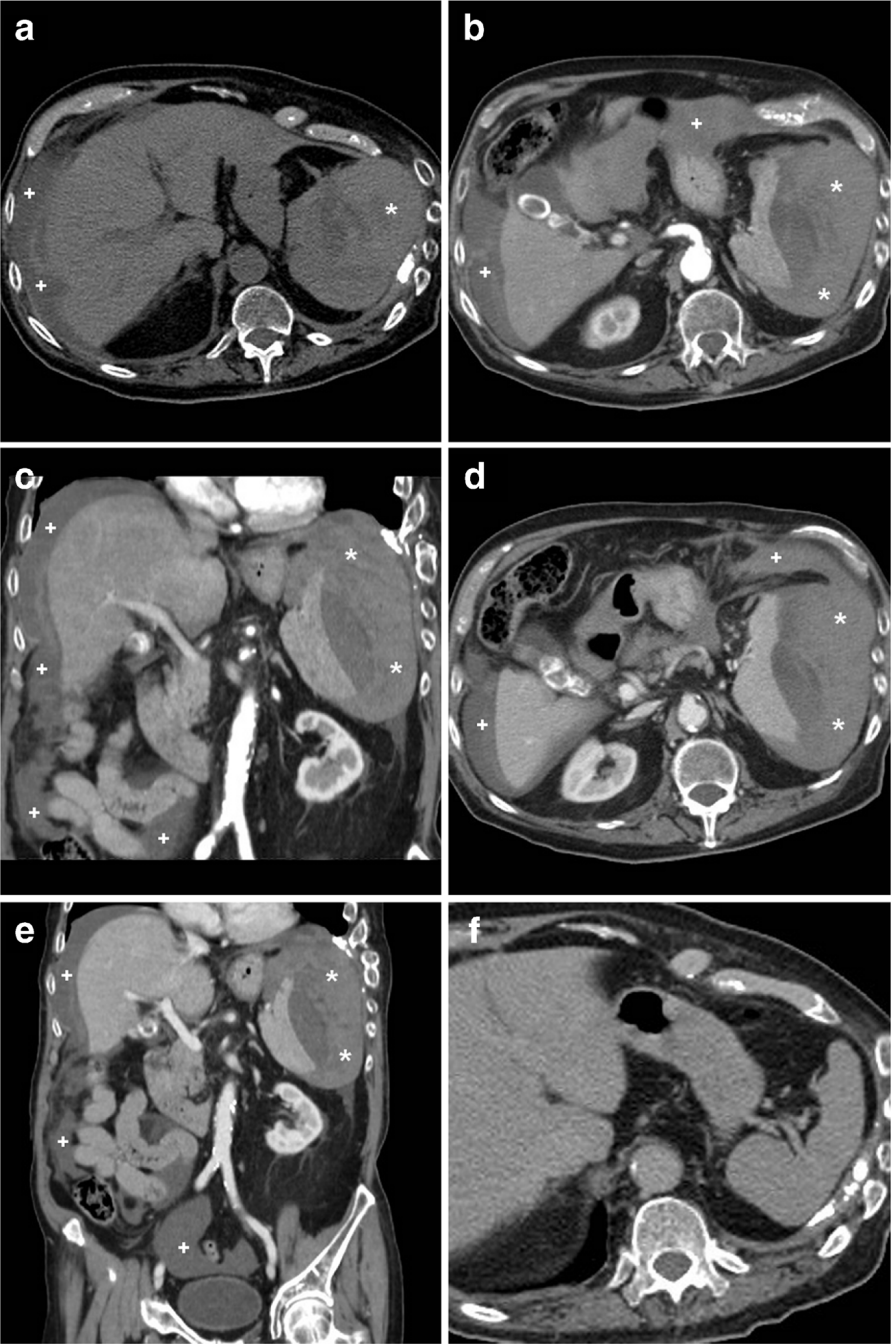
An elderly 89-year-old male with chronic heart failure, previous transurethral resection of non-muscle-invasive urinary bladder carcinoma, and lung emphysema experienced sudden hypotension and fainting. He was not on anticoagulants. Physical findings included palpation tenderness in the left hemiabdomen, tachycardia, and hypotension. Laboratory tests revealed severe blood loss: haemoglobin dropped from 9.2 to 6.4 g/dl within four hours. Platelet count, prothrombin time, and activated partial thromboplastin time were within normal range. Urgent CT including unenhanced (**a**), arterial- (**b**, **c**) and venous-phase (**d**, **e**) post-contrast images revealed mixed attenuation peritoneal effusion (+) consistent with haemoperitoneum. The spleen was surrounded, medially dislocated, and compressed by massive, fresh hyperattenuating (up to 55 Hounsfield units), partly subcapsular haemorrhage (*). Pseudoaneurysms and active bleeding were not seen. The spleen showed homogeneous parenchymal enhancement without focal lesions or signs of diffuse infiltrating disease. Retrospectively, contrast-enhanced CT obtained four months earlier (F) for bladder cancer staging revealed a normal, homogeneous spleen. The patient underwent urgent splenectomy and eventually recovered. Surgical pathology revealed medium-sized spleen with reactive lymphoid hyperplasia, and excluded acute infectious or neoplastic changes. Final diagnosis was idiopathic splenic rupture

Very uncommonly (7 % of all ASRs), “idiopathic” rupture may occur in a normal spleen (Fig. 2). The two hypothesised mechanisms involve: (a) intrasplenic cellular or reticuloendothelial hyperplasia leading to parenchymal engorgement and vascular occlusion, and (b) compression by the abdominal musculature during physiological activities such as sneezing, coughing, or defecation. Idiopathic ASR may be suggested when hemoperitoneum and high-grade splenic injury occur without CT imaging evidence of splenomegaly, focal masses, or splenic lesions. The consistent history is negative for recent trauma or surgery, known disease affecting the spleen, coagulopathy and anticoagulation, and signs and symptoms of systemic infection. The diagnosis is generally confirmed by negative viral serology and normal spleen at gross inspection and histology [4, 6–8].

In our opinion, general radiologists and emergency physicians should be well aware that splenic rupture with or without hemoperitoneum may occur in the absence of trauma and of previously diagnosed diseases involving the spleen. ASR should be strongly suspected when acute abdominal manifestations occur in young patients with acute infections, or in the setting of haemopoietic and lympho-reticular disorders. When anticoagulation and known systemic diseases are excluded, ASR is likely to be the manifesting complaint of an underlying infectious, immunologic, or neoplastic disorder: therefore, a quick but thorough history-taking and laboratory search for infections are required [2, 3, 7].

Early imaging diagnosis of ASR is warranted to limit mortality and to provide correct triage between “watchful waiting”, interventional, and surgical treatment. Ultrasound is a quick, noninvasive first-line technique to detect hemoperitoneum, which appears as complex hypoechoic effusion with regions of increased echogenicity. However, borrowing from experience in trauma, ultrasound has moderate sensitivity (72-78 %) for the detection of splenic rupture. Furthermore, sonographic evaluation may be limited by large body habitus and bowel gas, has limited specificity due to the variable echogenicity of abscesses and hematomas, and does not provide a panoramic investigation of the entire abdomen and pelvis [2, 3, 16, 17].

As correctly stated by Unal et al. [1], due to widespread availability and extreme acquisition speed, multidetector CT represents the ideal imaging modality to consistently assess patients presenting to the emergency department with acute abdomen and signs of hemodynamic instability. Since physical signs are often unclear and laboratory findings do not accurately reflect the entity of bleeding, CT is warranted to investigate suspected intra-abdominal bleeding [3, 12].

Depending on the patient’s haematocrit, recent extravascular blood measures at 35 to 60 Hounsfield Units (HU) of attenuation, and becomes even denser (60..80 HU) from clotting within a few hours (Figs. 1 and 2). The highest attenuation “sentinel clot” located nearest to the site of bleeding allows confident identification of the spleen as the injured organ. Furthermore, CT reliably allows detecting coexistent haemoperitoneum and ongoing bleeding, two features that are strongly associated with the risk of failed nonsurgical management. Haemoperitoneum (Figs. 1 and 2) is heralded by higher-than-water attenuation (30–45 HU) peritoneal effusion, often with a mixed appearance or fluid-fluid level. Noncontained active haemorrhage (Fig. 1) appears as serpiginous or jet-like extravasation of injected contrast medium, which follows the attenuation of blood vessels in all acquisition phases and generally progresses from the arterial to the venous phase. Finally, CT allows the differentiation of ASR from other rare non-traumatic causes of abdominal haemorrhage including ruptured liver (mostly hepatocellular adenoma or carcinoma rather than metastases) or kidney (particularly angiomyolipomas) tumours, visceral aneurysms and pseudo-aneurysms, and gynecologic conditions such as ectopic pregnancy, ruptured corpus luteum cysts, and HELLP syndrome [18–20].

Treatment guidelines for splenic injury from blunt abdominal trauma cannot be directly applied to ASR, since the latter commonly occurs in a diseased spleen and patients are generally older than those experiencing traumas. The correct therapeutic choice should consider the presence of haemodynamic instability, the amount of blood products used, the degree of haemoperitoneum, the underlying pathology, and the extent of splenic damage [21]. As is well known to radiologists who are familiar with polytrauma imaging, multidetector CT is crucial in this setting as it detects active bleeding, and reliably measures and categorises splenic injuries according to the American Association for the Surgery of Trauma (AAST) scale as either subcapsular and intraparenchymal hematomas, variably deep parenchymal and/or capsular lacerations, or devascularisation and fragmentation [22–25].

The majority (over 80 %) of reported ASR cases are treated surgically, and splenectomy remains the treatment of choice for patients with underlying malignancies. However, similarly to low-grade splenic traumas in haemodynamically stable patients, there is an increasing trend towards non-operative management for ASR as well, which achieves a high (80 %) success rate with correct patient selection. Conservative treatment including bed rest, intravenous fluids, and blood transfusions is particularly appealing in young and pediatric patients with acute infections, since preserving the spleen prevents long-term infectious morbidity. Strict clinical, laboratory, and imaging monitoring is required during nonoperative management: multidetector CT consistently allows assessment of changes in size and attenuation of haematomas over time [4, 5, 8, 16, 17]. Albeit operator-dependent and lacking panoramicity, the use of contrast-enhanced ultrasound allows younger patients to avoid irradiation from repeated CT studies [26].

**Fig. 3:**
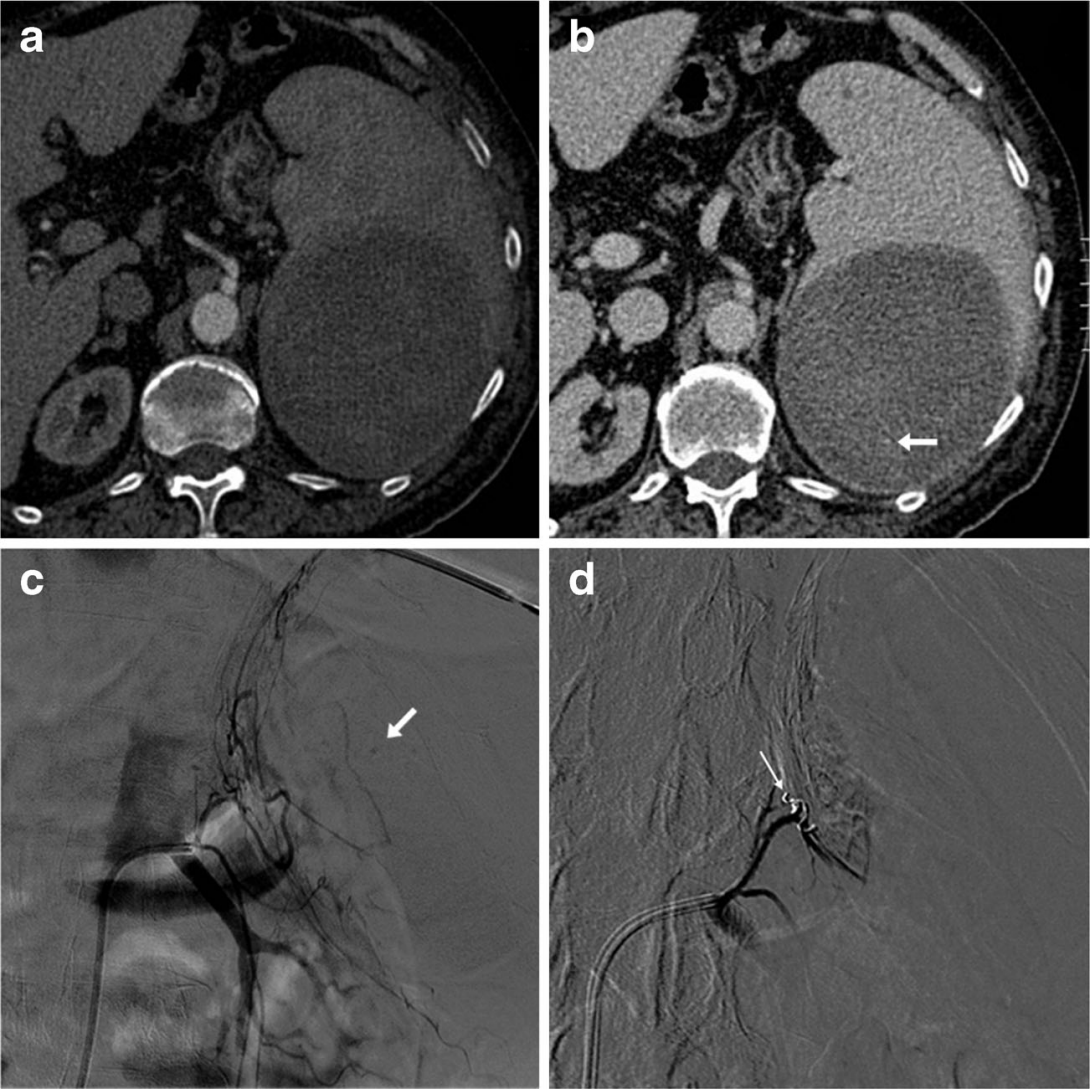
A 73-year-old male on anticoagulation suffered from spontaneous acute abdominal pain. His medical history included congestive heart failure, complete atrioventricular block treated with an implantable cardioverter-defibrillator, previous angioplasty and coronary stenting for acute myocardial infarction, and surgically treated colon carcinoma. Physical examination revealed hypotension and tender left hypocondrium. The international normalized ratio (INR) was 2.6; haemoglobin level was 6.5 g/dl. CT (**a**, **b**) revealed a large, roundish mixed attenuation intraparenchymal splenic haematoma without haemoperitoneum. Active bleeding was not evident during the arterial-phase acquisition (**a**). Faint contrast “blush” (arrow in **b**) was visible in the venous phase and confirmed at urgent arteriography as minimal bleeding originating from the left phrenic artery (arrow in **c**). Selective embolization with Spongostan and coil (thin arrow in **d**) was performed and allowed successful nonoperative management

Increasingly adopted to manage traumatic splenic injuries, interventional treatment with transcatheter arterial embolisation (TAE) may also prove a valuable non-surgical option for ASR (Fig. 3), particularly in cases associated with anticoagulation, malaria, and mononucleosis, and when CT detects active arterial bleeding [2, 17, 27, 28]. In selected patients, TAE is useful as a temporary stabilising measure. Combined with intensive care, interventional radiology may allow a more rapid and safer haemostasis than surgery alone [29].

The prognosis of ASR is generally related to the underlying disease. The non-negligible fatality rate approaches 15 % of patients. Risk factors associated with increased ASR-related mortality include splenomegaly, advanced age, and neoplastic disorders [8].
